# Location selection criteria for field hospitals: A systematic review

**DOI:** 10.34172/hpp.2022.17

**Published:** 2022-08-20

**Authors:** Keyvan Fardi, Ghader Ghanizadeh, Mohammadkarim Bahadori, Samaneh Chaharbaghi, Sayyed Morteza Hosseini Shokouh

**Affiliations:** ^1^Health Management Research Center, Baqiyatallah University of Medical Sciences, Tehran, Iran; ^2^Faculty of Health, Baqiyatallah University of Medical Sciences, Tehran, Iran

**Keywords:** Field hospital, Site selection, Systematic review, Meta-synthesis

## Abstract

**Background:** Establishing field hospitals is a critical task for governments to complete following disasters, with the first step being to identify suitable locations. Although field hospitals are similar to permanent hospitals and temporary shelters, no research in English has been conducted to extract the location selection criteria for field hospitals from those for hospitals and temporary shelters.

**Methods:** A meta-synthetic approach was used to review all related qualitative, quantitative, and mixed studies published in English between January 2010 and June 2020 to identify new field hospital site selection criteria distinct from those used to select a permanent hospital and temporary sheltering sites.

**Results:** From 4317 screened records, 24 articles were eventually identified as eligible studies, through which 151 open codes, 21 axial codes, and nine themes were identified. The top three axes included proximity to main roads, proximity/distance to permanent hospitals, and proximity/distance to other medical centers.

**Conclusion:** By considering a field hospital as a facility with certain characteristics similar to permanent hospitals and temporary shelters, it is possible to identify specific new criteria and sub-criteria by extracting items common to permanent hospitals and temporary shelter site selection studies.

## Introduction

 Improving human well-being and facilitating access to convenient, affordable, and high-quality health care services are among all countries’ primary concerns. As the primary providers of medical treatment services, permanent hospitals play a pivotal role in health care systems.^1^ Under normal circumstances, permanent hospitals provide a certain level of medical care to communities. However, a disaster can devastate some permanent hospitals’ capacity. Additionally, following a disaster, such as an earthquake, local medical facilities see an increase in demand (injured people). As a result, policymakers should contemplate the establishment of temporary medical facilities, such as field hospitals.

 Even if all acute emergency beds in medical centers were available following a catastrophic event, additional beds would be required to accommodate extra referrals to medical facilities. A field hospital is a self-contained, ambulatory medical facility that can be rapidly deployed and expanded to meet emergency medical needs. Regardless of its simple definition, establishing and operating a field hospital is a costly endeavor. Thus, authorities and policymakers must place a premium on determining the most appropriate location(s) for a temporary medical unit.^2^

 A temporary shelter is one type of facility that may resemble a field hospital in terms of usage. Three steps can be taken to establish these spaces: emergency, temporary, and permanent. Following the evacuation process, actual or potential disaster victims will spend a brief period in an emergency shelter before being relocated to temporary housing until their permanent locations are ready.^3^ Permanent hospitals, as prefabricated structures and the most well-known providers of medical services, are critical facilities on which disaster managers rely during the response phase. As with temporary shelters, these medical centers have their own set of site selection criteria, not all of which overlap with those of field hospitals. Compared to the numerous studies that propose and evaluate emergency site selection criteria for temporary shelters and permanent hospitals, there are few published articles on field hospital site selection. Nonetheless, permanent hospitals and temporary shelters share some characteristics in terms of site selection.

###  Purpose and research questions

- Defining critical site selection criteria for permanent and field hospitals.
- Developing similar site selection criteria for hospitals, field hospitals, and temporary shelters.
- Extrapolating essential site selection criteria from permanent hospital and temporary shelter site selection and incorporating them into field hospital site selection.

## Materials and Methods

 A qualitative meta-synthesis based on Sandelowski and Barroso^4^ was conducted to define the most critical field hospital site selection criteria clearly. By enabling researchers to identify, differentiate, and evaluate published studies on field hospitals, permanent hospitals, and site selection for temporary sheltering, this qualitative review technique enables a novel interpretation of the data, thereby identifying additional research gaps that can be addressed in future research. Using Sandelowski and Barroso^4^ as a guide, the following steps comprise this study: defining the research’s purpose and posing pertinent questions, selecting the studies that are most pertinent to the research questions, systematically reviewing the collected papers, extracting valuable information, and creating data charts, evaluating the reviewed studies, and finally, proposing a thematic analysis based on the findings.

###  Searching and retrieving qualitative research 

 To investigate the triple streams of this study, the most significant keywords identified were *hospital location*, *temporary sheltering location*, and *field hospital*. However, additional terms were considered to broaden the research domain, such as *red crescent location*, *red cross location*, and *temporary medical centers*. Almost all keywords included the word *location* to help specify and narrow the search area. Science Direct, Springer, PubMed, and Google Scholar were the electronic databases from which the systematic review articles were extracted based on the PRISMA^5^ protocol. Due to technological advancements, previous location selection criteria were deemed outdated for modern use; thus, the search period was limited to the last decade (from January 1, 2010 to June 1, 2020).

**Figure 1:**
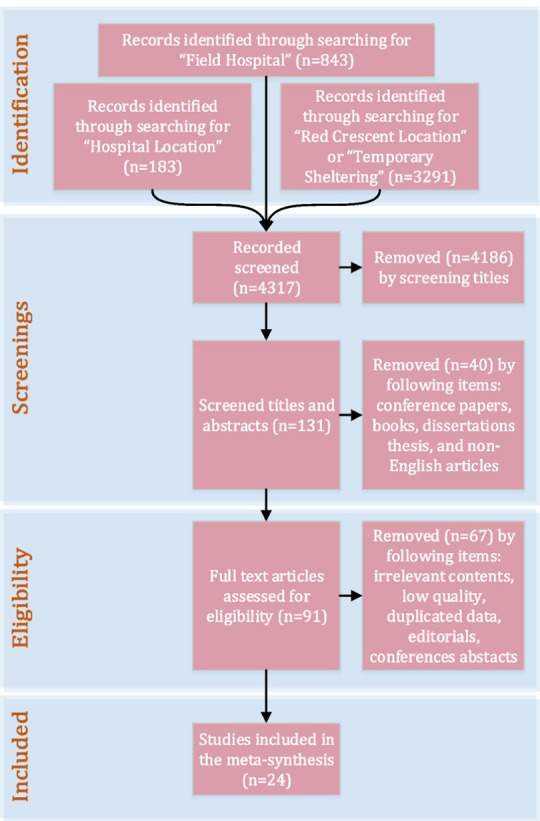
The PRISMA flowchart

 Additionally, essential studies were selected for the meta-synthesis from published articles, excluding unpublished work or dissertations. After screening the titles of 4317 articles, the abstracts of 131 studies were reviewed. There were 24 articles that met the inclusion/exclusion criteria (Figure 1). Although the final number of articles selected was significantly less than anticipated, these articles contained valuable information regarding predefined research questions. The selected studies addressed field hospital location, hospital location, temporary shelter location, red crescent camp location, and red cross camp location following an earthquake. More precisely, five articles (n=5) discussed field hospital locations, six (n=6) discussed hospital locations, and 13 (n=13) discussed temporary shelter locations and other similar locations.

###  Appraising qualitative research reports

 Two assistants worked under the direction of the research team leader to collect and analyze related articles using the virtual platform. Afterward, two research assistants independently screened the most closely linked articles about field hospitals, permanent hospitals, and temporary shelter site selection published between 2010 and 2020 based on their abstracts and titles. The inclusion criteria were related to locations and time horizons. Subsequently, exclusion criteria (books, dissertations, non-English, or low-quality articles) were applied, and a final selection of articles was obtained. The authors communicated regularly throughout the inclusion and exclusion phases to ensure the research’s validity, accountability, and reliability. All of the studies specifically mentioned earthquakes as a cause of at least one of the disasters.

 The first and third authors oversaw the quality appraisal process as the meta-synthetic stages progressed, under the leadership of the second author. The second author supervised the first author throughout the process of reviewing and coding (open and axial) all 24 articles to gain a thorough understanding of their contents, followed by the extraction of the essential items. MAXQDA 2018 was used to code the selected articles as a qualitative data analyzer, identifying repeated items, familiar concepts, and emerging themes. The first and third authors carried out the task of comparing similar and dissimilar items with close consultation with the second author. The group then discussed the rational relationships that existed between the data extracted from the articles. Regular meetings were held to discuss the study’s limitations, identify novel items, and introduce new avenues for future research. To the extent possible, articles included were from the first quarter or had a high impact factor. However, due to a dearth of published articles on site selection for field hospitals, several of the articles considered did not fully meet the conditions outlined.

## Results

###  Classifying findings in qualitative research reports

 After developing the research question, conducting the systematic review, and locating appropriate texts, pertinent information was extracted from the selected articles, and the final 24 articles in Table 1 underwent the open coding process.

**Table 1:** Developing open codes (sub-criteria)

**Author**	**Case Study**	**Group**	**Type of Research**	**Identified Codes**
Tai et al^6^	Taiwan	TSL	Descriptive, classification and mixed	Proximity to secondary road, Proximity to residential zone, Proximity to police station, Proximity to main road, Proximity to fire station
Paul and Hariharan^7^	USA	TSL	Descriptive and quantitative	Topography of the terrain, Proximity/Distance to permanent hospitals, Proximity to main road, Distance to fault lines
Burkey et al^8^	USA	HL	Descriptive and quantitative	Proximity/Distance to permanent hospitals, Proximity to potential patients
Chatterjee and Mukherjee^9^	India	HL	Descriptive and mixed	Proximity to market, Space for future construction, Population Density Construction and maintenance costs, Economic condition, Topography of the terrain, Land ownership, Land price, Proximity to Public transportation Facility Availability
Chen et al^10^	China	TSL	Descriptive and quantitative	Proximity to main road, Distance to River, Distance to Power lines, Distance to Natural gas reserve station, Distance to Natural gas gate station. Distance to Historic relics, Distance to Gas station, Accessibility to sewage treatment system, Access to Power station.
Vafaei and Öztayşi^11^	Turkey	FHL	Descriptive and quantitative	Proximity to main road, Population Density, Capacity of Beds, Proximity/Distance to permanent hospitals
Kılcı et al^12^	Turkey	TSL	Descriptive and quantitative	Type of terrain, Topography of the terrain, Slope of the terrain, Proximity/Distance to permanent hospitals, Proximity to market, Proximity to main road, Land ownership, Flora of the terrain. Distance to Warehouses, Accessibility to sewage treatment system, Accessibility to electrical Infrastructure
Sadidi et al^13^	Iran	TSL	Descriptive and quantitative	Slope of the terrain, Proximity/Distance to other medical centers, Proximity to secondary road, Proximity to main road, Proximity to fire station, Distance to Power lines, Distance to fault lines
Nappi and Souza^14^		TSL	Classification and qualitative	Proximity/Distance to other medical centers, Proximity to transport facilities, Proximity to secondary road, Proximity to school, Proximity to recreational centers, Proximity to police station, Proximity to market. Proximity to main road, Proximity to local primary storage, Proximity to humanitarian aid suppliers, Proximity to food distribution sites, Proximity to affected areas, providing thermal comfort, Population Density, minimizing direct sun exposure, Manmade Dangerous Zones, Land use, Having ventilation system, Expenses on adjustments, Construction and maintenance costs, Compatibility, Availability of power source, Availability of natural ventilation, Authority permission, Adaptation to climate change, Accessibility to worship places, Accessibility to sewage treatment system. Accessibility to sanitary system, Accessibility to provisions for affected people, Accessibility to airport/airfields/helipads
Hosseini et al^15^	Iran	TSL	Descriptive and mixed	Type of plants, Topography of the terrain, Slope of the terrain. School capacity of neighborhood, Quality of medical care services of neighborhood, Proximity/Distance to other medical centers, Proximity to police station, Proximity to material resource center, Proximity to landfill sites, Proximity to green space, Proximity to fire station, Preparation costs. Population density of neighborhood, Population Density, Manmade, Dangerous Zones, Level of groundwater, Land use, Land price, Land ownership, Firmness of soil, Distance to natural protected areas, Adverse environmental impact, Accessibility of emergency services to the location Accessibility of departures people to other part of the city
Xu et al^16^	China	TSL	Descriptive and mixed	Slope of the terrain, Proximity to main road, Proximity to affected areas. Distance to risky buildings, Distance to lakes, Distance to Gas station, Distance to fault lines, Distance to chemical factories, being wide open space, being stadiums, being squares, being schools, being park. being leisure/entertainment place, being large enough, being green space, Accessibility to safe water, Accessibility to electrical Infrastructure. Anti-seismic Quality
Eldemir and Onden^17^	Turkey	HL	Descriptive and mixed	Proximity to recreational centers, Proximity/Distance to other medical centers, Proximity to business area, Proximity to educational facilities. Proximity to residential zone, Proximity to main road, Proximity to Public transportation, Proximity to subway, Proximity to railway station. Proximity to seaport
Khodaparasti et al^18^	Iran	HL	Descriptive and quantitative	Proximity to potential patients, Proximity to affected areas
Dell’Ovo et al^19^	Italy	HL	Descriptive, classification and mixed	Proximity to library, Proximity/Distance to permanent hospitals. Proximity to post office, Proximity to restaurant, Proximity to school. Accessibility to worship places, Proximity to fire station, Potential of the area to become an attractive pole, Reuse of built-up area, Population Density, Demand, Distance to air polluted area, Distance to noise polluted area, Land contamination, Land size, Land price, Land ownership. Proximity to green space, Distance to River, Proximity to transportation hubs, Proximity to Public transportation, Accessibility to sewage treatment system
Moradian et al^20^		HL	Classification and qualitative	Accessibility to drainage system, Accessibility to electrical Infrastructure... Accessibility to sewage treatment system, Community health status Convenience of traffic, Distance to fault lines, Distance to industrial centers. Distance to River, Economic constrains, Governmental regulations. Healthcare spending per household, Interested and affected people, Land price, Number of patients rejected by hospitals, Number of visits to doctors. Patient transfer rates, Policymaker and key persons’ attitude, Population age distribution, Population Density, Proximity to green space. Proximity to main road, Proximity to population at risk, Proximity to Public transportation, Proximity to railway station, Proximity to subway, Proximity/Distance to permanent hospitals, Social inconvenience, Socio economic status of the population, Space for future construction. Temporal restriction, Urban planning
Trivedi and Singh^21^	Nepal	TSL	Descriptive and mixed	Type of terrain, Topography of the terrain, Slope of the terrain, Proximity\ Distance to other medical centers, Proximity to market, Proximity to affected areas, Probability of Seismic activity, Flora of the terrain, Distance to Warehouses, Density of affected population, Accessibility to sanitary system, Accessibility to electrical Infrastructure, Anti-seismic Quality
Pouraliakbarimamaghani et al^22^		FHL	Descriptive and quantitative	Distance to warehouses, population density, proximity/distance to permanent hospitals
Moradian and Ardalan^2^		FHL	Classification and qualitative	Proximity to main road, accessibility to airport/airfields/helipads, proximity to railway station, proximity to seaport, distance to other high risk area. Socio economic status of the population, economic constrains. Community health status, population age distribution, risk level of existing hospitals, population density, governmental regulations, policymaker and key persons’ attitude, Be the capital of the province, Proximity/distance to other medical centers, number of permanent hospitals
Trivedi^23^		TSL	Classification and mixed	Topography of the terrain, Slope of the terrain, Road condition, Proximity/Distance to other medical centers, Proximity to transportation hubs, Proximity to school, Proximity to recreational centers, Proximity to material resource center, Proximity to market, Proximity to main road, Proximity to landfill sites, Presence of trees, Manmade Dangerous Zones, Land ownership, Flora of the terrain, Firmness of soil, Distance to Warehouses. Convenience of traffic, Accessibility to worship places, Accessibility to telecommunication facilities, Accessibility to safe water, Accessibility to local economic services, Accessibility to electrical Infrastructure. Accessibility to drainage system, Accessibility to Burial/cremation sites Accessibility to airport/airfields/helipads
Boostani et al^24^	Iran	TSL	Descriptive and mixed	Proximity to fire station, Proximity to police station. Proximity/Distance to permanent hospitals, Proximity to school, Proximity to market, Proximity to recreational centers, Proximity/Distance to other medical centers, Accessibility to sports halls, Distance to touristic areas. Capacity expansion, Proximity to affected areas, Construction and maintenance costs, Acceptability by local people, Distance to trees and vegetation cover, Distance to habitat of endangered species, Distance to natural protected areas, Proximity to floodplains and wetlands. Distance to urban polluted areas, Proximity to clean energy resources. Climatic conditions of the region, Slope of the terrain, Land ownership. Land use, Land price, Accessibility to parks, Distance to fault lines. Distance to Gas station, Manmade Dangerous Zones, Landslide, Distance to high voltage transmission lines, Distance to gas transfer lines. Distance to refineries, Quality and reliability, Proximity to main road. Accessibility to airport/airfields/helipads, Proximity to humanitarian aid suppliers, Proximity to railway station, Proximity to transportation hubs. Accessibility to sewage treatment system, Access to Power station. Accessibility to telecommunication facilities, Accessibility to drainage system, Availability of power source, Accessibility to energy resources. Accessibility to safe water
Nappi et al^25^	Brazil	TSL	Descriptive & Classification & Mixed	Proximity to workplace, Proximity to school, Proximity to market, Proximity to recreational centers, Accessibility to worship places, Proximity/Distance to other medical centers, Being wide open space. minimizing direct sun exposure, Proximity to population at risk, Acceptability by local people, provide protection from the weather, Availability of natural ventilation, environmentally sustainable, Distance to trees and vegetation cover, Manmade Dangerous Zones, Proximity to humanitarian aid suppliers, Accessibility to sewage treatment system. Accessibility to safe water, Accessibility to energy resources
Song et al^26^	China	TSL	Descriptive and mixed	Proximity to recreational centers, Governmental regulations, Local culture. Construction and maintenance costs, Adverse environmental impact. Topography of the terrain, Proximity to transport facilities. Proximity to material resource center, Proximity to landfill sites
Gai et al^27^	China	FHL	Descriptive and mixed	The timeliness of communication, firmness of soil, facility availability. convenience of traffic, accessibility to safe water
Oksuz and Satoglu^28^	Turkey	FHL	Descriptive and quantitative	Road condition, Proximity/Distance to permanent hospitals, proximity to affected areas, possibilities of damage to the hospitals, casualty capacities of permanent medical centers

Abbreviations: TSL, temporary sheltering location; HL, hospital location; FHL, field hospital location.

###  Synthesizing research findings 

**Figure 2:**
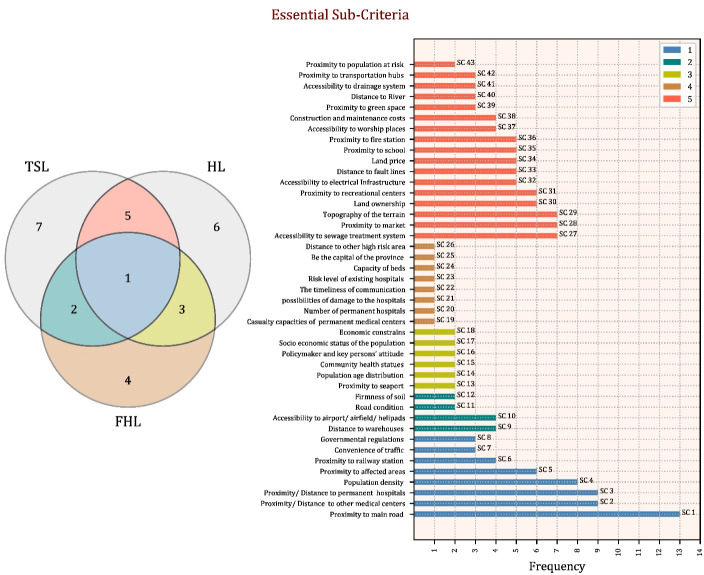
Sorted sub-criteria (TSL: temporary sheltering location; HL: hospital location; FHL: field hospital location)

**Figure 3:**
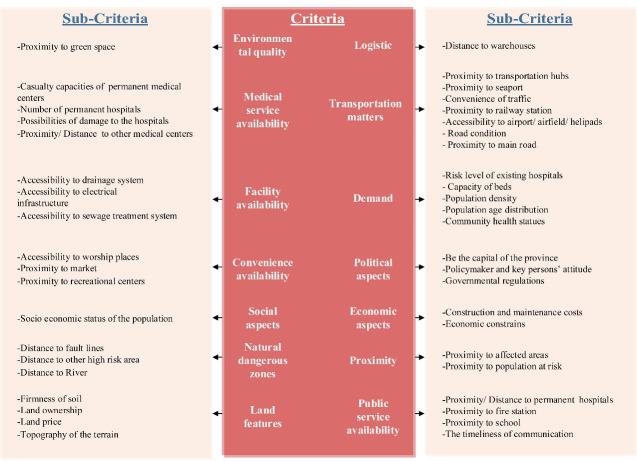
Developing criteria form sub-criteria

**Figure 4:**
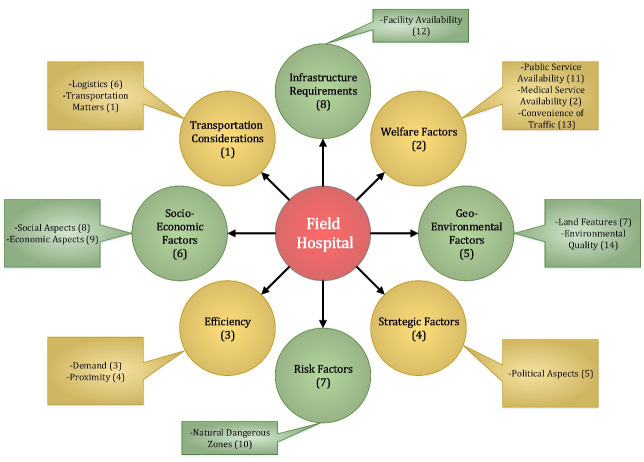
Conceptual diagram of themes and their related axial-codes

 The majority of the studies are represented as open codes in Table 1. As a result of the primary open coding process, 196 items were identified. Then, similar items were merged, reducing the number of open codes to 151. We reduced open codes that were more significant for hospital site selection and temporary shelter site selection following consultation with group members, resulting in 43 open codes (see Figure 2). Afterward, open codes were classified into 14 categories based on their similarities (see Figure 3). Finally, these criteria were subdivided into eight distinct categories (see Figure 4).

## Discussion

 The authors conducted this review article based on a meta-synthetic approach due to the lack of a comprehensive study examining the similarity of site selection between hospitals, temporary shelters, and field hospitals. A systematic review approach identified 24 articles. Then, using the synthesizing process, 151 open codes, 21 axial codes, and nine themes were obtained. Finally, prioritization algorithms revealed 43 open codes, 14 axial codes, and eight themes. To the best of the authors’ knowledge, 15 open codes that were not previously considered Field Hospital Location Selection (FHLS) sub-criteria were identified. These open codes included proximity to transportation hubs, accessibility to places of worship, proximity to markets, proximity to recreational centers, proximity to fire stations, proximity to schools, proximity to vulnerable populations, land ownership, land value, terrain topography, proximity to green space, construction and maintenance costs, distance to fault lines, distance to rivers, accessibility to drainage systems, accessibility to electrical infrastructure, and accessibility to sewage treatment systems.

 For hubs, open codes included accessibility to places of worship, proximity to markets, proximity to recreational centers, proximity to fire stations, proximity to schools, proximity to vulnerable populations, land ownership, land value, terrain topography, proximity to green space, construction and maintenance costs, distance to fault lines, distance to rivers, accessibility to drainage systems, accessibility to electrical infrastructure, and accessibility to sewage treatment systems. As implied by their titles, while most of these 43 open codes, such as proximity to market, do not require additional explanation or discussion, the remainder, such as the terrain’s topography, do. In this regard, the following section proposes interpretations and arguments for these open codes:

###  Transportation considerations 

 Several reasons why researchers believe this item is necessary are: (1) In cases of temporary shelter site selection, large numbers of displaced individuals can be easily transferred to shelters. (2) When it comes to hospital site selection, individuals can easily access these medical facilities. (3) Through site selection for field hospitals, victims suffering from acute injuries can be transferred more quickly to permanent hospitals for more specialized care. (4) These arterial roads serve as an effective logistical support network in all cases. Proximity to transportation hubs is a novel sub-criterion identified in the identified transportation open codes. While proximity to railway stations and seaports (both of which are examples of transportation hubs) is mentioned separately, proximity to transportation hubs is also proposed to encompass other types of transportation hubs in the affected area.

###  Welfare factors

 As previously stated, certain sub-criteria are defined somewhat ambiguously: proximity/distance to other medical centers and proximity/distance to permanent hospitals as open codes within medical service availability. As illustrated in Figure 1, these two items were mentioned in all three study streams. According to studies on site selection for temporary shelters, proximity to medical centers and hospitals increases the attractiveness of candidate locations.^7,12,24^ On the other hand, the competition factor must be considered when it comes to hospital site selection, as hospitals prefer to be located as far away from other hospitals and medical centers as possible.^8,19,20^ From the perspective of field hospital site selection, proximity to hospitals and other permanent medical facilities can be viewed positively or negatively. According to Vafaei and Öztayşi,^11^ proximity to hospitals enables medical teams to transfer patients to permanent locations for additional treatment processes quickly. Conversely, being isolated from other medical facilities enables authorities to cover a larger area. Thus, this factor should be viewed as a trade-off between increasing overall coverage by medical centers (temporary or permanent) in the region and decreasing patient transfer times between permanent medical centers and field hospitals.

###  Efficiency

 According to Figure 2, a study about field hospital site selection mentions the risk level of existing hospitals. This item prompts risk managers to consider the infrastructural state of existing hospitals in the aftermath of destructive earthquakes under various scenarios. Not only would permanent hospitals be rendered inoperable in the event of widespread destruction, but their patients would have to be transferred to other functioning medical facilities. As a result, proximity to hospitals with sturdy structures is critical. In other instances, a hospital with a robust structure may be able to provide services to injured residents. As a result, field hospital locations should be relatively distant from or closer to existing permanent hospitals, depending on their structural integrity. Additionally, decision-makers must consider the number of beds in permanent hospitals, population density, age distribution, and community health statutes to maximize efficiency following disasters. Finally, in addition to proximity to the affected area, proximity to the vulnerable population was identified as a novel sub-code for field hospital site selection, requiring policymakers to locate field hospitals closer to potentially vulnerable people living in less robustly constructed urban areas.

###  Strategic factors

 While foresight, unattractiveness, and political aspects are all axial codes for this theme, only the last is identified as a critical criterion for field hospital location selection. Government regulations (as the most effective sub-criterion) emphasize the critical role of government financial assistance and incentives in establishing field hospitals throughout a region. While the articles reviewed did not detail the precise relationships between policymakers and service providers, it appears that this open code has a close relationship with government regulations. However, the current study treats these sub-criteria differently. Finally, locating field hospitals in the provincial capital facilitates access to critical assets and logistical support.

###  Geo-environmental factors

 With eight open codes, environmental concerns are not considered critical factors in determining the location of field hospitals. As a result, Figure 3 depicts only land features and environmental quality as essential criteria for this theme. The first unsolved code of this theme that requires explanation is the soil’s firmness. This means that areas with harder soils are more advantageous because they are more resistant to flooding and landslides.^23,27^ The next item is land ownership, classified into two categories: private and public.^19^ Obviously, government-owned land enables disaster managers to begin rescue operations as quickly as possible.^23^ To minimize overall costs, particularly land costs, areas with lower prices or even free use must be considered. Thus, managers should seek out government-owned land.^24^ Seven articles discussed the terrain’s topography as a significant element. Kara, and Bozkaya^12^ provide a comprehensive explanation of this open code, stating that the most desirable locations for establishing temporary shelters are savannah, valley, piedmont, and stream beds.

###  Socioeconomic factors

 This theme is divided into two categories: social and economic. Two studies suggest that the theme’s first axis should be the population’s socioeconomic status. The effect of this sub-criteria, on the other hand, is not adequately explained. Thus, several external studies are being conducted to determine the effect of socioeconomic status on the necessity of establishing field hospitals. According to Epstein et al^29^ poor people require a higher level of medical care. Additionally, Moore et al^30^ assert that the average length of stay in impoverished areas is longer than in prosperous areas. As a result, it is reasonable to conclude that field hospitals should be established closer to areas with lower socioeconomic status. Economic constraints are the next open code in this theme, which has received scant attention in the literature. However, it is clear from the extracted codes that this sub-criterion is closely related to governmental regulations, policymakers’ and key stakeholders’ attitudes, landowners’ attitudes, and land prices. Finally, due to the temporary nature of field hospitals, construction and maintenance costs (as a novel field hospital site selection criteria) can be reduced to only construction costs, including labor and material costs, among others.

###  Risk factors

 According to Table 1, five articles emphasized the importance of distance to fault lines. These studies recommended locating temporary shelters and hospitals as far away from fault lines, rivers, and other high-risk areas as possible. Sadidi et al^13^ recommended that temporary shelters be located at least 200 m from fault lines and 50 m from rivers. Field hospitals must be established as far away from danger zones as possible. There is, however, an exception. Given that the communities served by field hospitals are earthquake victims, it seems logical to locate field hospitals near fault lines, rivers, or other high-risk areas. Indeed, proximity to these fault lines, rivers, and other high-risk areas enhances the service levels of these temporary medical centers, as these areas have a greater potential for fatalities and injuries.^31^

 As illustrated in Figure 4, the natural danger zone is the sole criterion for determining the risk factor. However, both temporary sheltering and hospital site selection studies address the various open codes of manufactured danger zones. For example, Chen et al,^10^ Xu et al,^16^ and Boostani et al^24^ mention the distance to gas stations in their articles on temporary shelter site selection. Furthermore, Moradian et al,^20^ in a hospital site selection article, represent the distance to industrial centers (as an open code of manufactured danger zones). Thus, when selecting appropriate field hospital locations, taking this criterion into account can increase the location’s reliability. Because additional axial codes (such as environmental concerns, unattractiveness concerns, foresight, and accessibility) are only mentioned in connection with temporary sheltering or hospital site selection (such as environmental concerns, unattractiveness concerns, foresight, and accessibility), they may have little effect on field hospital location selection.

###  Infrastructure requirements

 As illustrated in Figure 3, this theme has two axial codes: facility availability and resource availability. According to the applied prioritization process, only facility availability is required for field hospital location selection. This criterion is subdivided into three sub-codes. Access to sewage treatment systems is the most critical of these requirements. According to this open code, the selected site should be located at a practical distance from these sewage systems to avoid potential hygiene issues. For example, Chen et al^10^ suggest a distance of 100 m from these systems; the next item is infrastructure accessibility. Since electrical equipment in a field hospital requires power, these facilities are equipped with generators to provide adequate energy. On the other hand, access to electrical infrastructure can be viewed positively.

###  Favorability of prebuilt locations

 Among the mentioned themes, it is clear that only the favorability of prebuilt locations is not considered a critical factor in field hospital location selection. However, the possibility of considering these types of locations during the site selection process for field hospitals has been discussed. Bar-Dayan et al^32^ discussed the establishment of an Israeli Field Hospital in Turkey’s Marmara region. The authorities initially planned to establish the Israeli Field Hospital inside the Turkish Ministry of Forestry building but later realized that the structure was at risk of collapsing due to aftershocks. As a result, neither the patients nor the crew desired to remain inside the building. However, they ultimately decided to relocate the Israeli Field Hospital to the ministry’s garden, with the operating theater remaining inside the structure. As a result of this experience, it appears prudent to consider prebuilt locations as potential field hospital sites. However, the location’s safety conditions must also be considered in advance. Finally, while the field hospital’s water supply should be located outside the disaster zone, having access to a well-designed drainage system can help reduce the risk of water contamination and infectious disease transmission among field hospital personnel and patients.

###  Strengths and limitations

 Several of the current study’s strengths include the following: precise and clear research questions; a rigorous and robust search strategy based on data mining from multiple databases; and reporting search results using the PRISMA^5^ flowchart. Additionally, the open codes analyzed in this study are well-defined items in the final study selection, which enables the authors to present them here with little risk of encroachment or overlap. Additionally, MAXQDA software helps classifying qualitative data, indexing them effetely, and removing duplications. Furthermore, using Sandelowski and Barroso^4^ methods enables the optimal synthesis of open and axial codes to achieve the final themes. Finally, combining these three study streams resulted in identifying previously unknown field hospital site selection criteria.

 Nonetheless, the current study’s design has some limitations and weaknesses. The language constraint is the primary constraint, as other studies on field hospital site selection may be conducted in other languages. The next limitation is the exclusive focus on articles, as only published articles met the inclusion criteria. There may be pertinent dissertations and theses not published in peer-reviewed journals. Another limitation is that the inclusion and exclusion criteria are solely based on seismic activity. On the other hand, field hospitals have a broader range of applications, including battlefields and other disaster situations. Moreover, additional research may wish to broaden the time span of the studies reviewed.

## Conclusion

 Despite the critical role of field hospitals in disaster management, few studies have examined how these temporary medical centers are located. We believe that field hospitals’ site selection is similar to that of temporary shelters and hospitals. The findings indicate that 26 sub-criteria are comparable between field hospitals and temporary sheltering or hospitals. Furthermore, we identified 17 sub-criteria critical for establishing temporary sheltering and hospitals that are not used in the site selection process for field hospitals. When establishing field hospitals, access to a sewerage treatment system, proximity to a market, and the train’s topography are the top three considerations for policymakers.

## Authors’ contributions

 KF, Conceptualization, literature search, methodology, data curation, appraisal, formal analysis, software, visualization, writing – original draft, writing – review & editing. GG, Conceptualization, critical appraisal. MB, Critical appraisal. SC, Literature search. SMHS, Conceptualization, major supervisor, methodology, critical appraisal.

## Funding

 No funding to declare.

## Ethical approval

 This research was performed based on Baqiyatallah University of Medical Sciences ethics committee approval (Approval ID: IR.BMSU.REC. 1398. 188).

## Competing interests

 There is no competing interest.

## Disclaimer

 No part of this paper is copied from other source.
